# Serum Concentration of Bone Metabolism Biomarkers in Goats during the Transition Period

**DOI:** 10.1155/2020/4064209

**Published:** 2020-01-31

**Authors:** Mohamed Tharwat

**Affiliations:** ^1^Department of Veterinary Medicine, College of Agriculture and Veterinary Medicine, Qassim University, P.O. Box 6622, Buraidah 51452, Saudi Arabia; ^2^Department of Animal Medicine, Faculty of Veterinary Medicine, Zagazig University, Zagazig, Egypt

## Abstract

**Objective:**

During the transition period, the animal experiences a series of nutritional, physiological, and social changes. The objective of the present study was to evaluate the influence of the periparturient period in goats on the serum concentrations of the bone biomarkers osteocalcin (OC), bone-specific alkaline phosphatase (b-ALP), and pyridinoline cross-links (PYD).

**Method:**

Blood samples were collected from fifteen female goats during the periparturient period 3 wk before expected parturition (*T* −3), within 12 h of parturition (*T* −3), within 12 h of parturition (*T* −3), within 12 h of parturition (

**Results:**

Compared to a value of 77.67 ± 47.6 ng/mL at *T* −3), within 12 h of parturition (*T* −3), within 12 h of parturition (*T* −3), within 12 h of parturition (*T* −3), within 12 h of parturition (*T* −3), within 12 h of parturition (*T* −3), within 12 h of parturition (*P* > 0.05). Compared to a value of 42.00 ± 19.50 U/L at *T* −3), within 12 h of parturition (*T* −3), within 12 h of parturition (*T* −3), within 12 h of parturition (*T* −3), within 12 h of parturition (*T* −3), within 12 h of parturition (*T* −3), within 12 h of parturition (*P* > 0.05). Compared to a value of 42.00 ± 19.50 U/L at *T* −3), within 12 h of parturition (*T* −3), within 12 h of parturition (*P* > 0.05). Compared to a value of 42.00 ± 19.50 U/L at *T* −3), within 12 h of parturition (*T* −3), within 12 h of parturition (*P* > 0.05). Compared to a value of 42.00 ± 19.50 U/L at

**Conclusion:**

The results of this study showed that the bone formation biomarkers (OC and b-ALP) did not change significantly during the periparturient period, while the bone resorption biomarker decreased significantly at parturition compared to 3 wk before and 3 wk after parturition. The significantly increased serum estrogen around parturition may have had a role in the increased bone resorption at this time.

## 1. Introduction

The transition period, 3 wk before to 3 wk after parturition, is characterized greatly by the increased risk of disease [1–3]. During this period, the animal experiences a series of nutritional, physiological, and social changes, and is more vulnerable to infectious and metabolic diseases [4]. Metabolically, the animal is in a state of nutrient reserve mobilization, primarily that of adipose and labile protein but also that of bone [1]. Successful transition of the animal from the fetal to the neonatal state involves tremendous physiological adaptations on the part of the neonate and the dam. The success or failure of this transition process equally dictates the survival of the offspring and the subsequent recovery of the dam [1, 4].

The common biomarkers of bone formation include osteocalcin (OC), bone-specific alkaline phosphatase (b-ALP), and amino and carboxy propeptides of collagen type I. The most common biomarkers of bone resorption on the other side include pyridinoline cross-links (PYD), deoxypyridinoline enzyme, tartrate-resistant acid phosphatase, and amino and carboxy telopeptides of collagen type I. In human medicine clinical practice, biochemical markers of bone turnover are widely used, mainly for noninvasive monitoring of bone metabolism and response to therapy of certain musculoskeletal and bone disorders [5, 6]. In veterinary medicine, bone biomarkers are mostly used in preclinical and clinical studies as a rapid and sensitive method for assessment of bone response to medical treatment and surgical interventions and for the detection of musculoskeletal injuries [7–10]. The use of bone biomarkers appears to be valuable for evaluating the bone remodeling status in cows and mares during the periparturient period [11–14].

This study was carried out to evaluate the effect of the periparturient period on the serum concentration of bone metabolism biomarkers in goats. Studying bone metabolism biomarkers during the transition period in goats is expected to increase our understanding to the biology of reproduction in this species.

## 2. Materials and Methods

### 2.1. Animals and Ethical Approval

The experimental protocol was approved by the Animal Ethical Committee, Deanship for Scientific Research, Qassim University, Saudi Arabia. The experimental design has been described previously [15, 16]. Briefly, fifteen dairy nonpregnant does (age, 22.6 ± 8.7 months; weight 42.3 ± 6 kg), reared at Qassim University Farm, were synchronized by inserting the controlled-release internal drug EAZI-BREED CIDR (Pfizer, Auckland, New Zealand), containing 0.3 g progesterone, into the vagina for 12 d. At the time of CIDR removal, each doe received an injection of 600 IU equine chorionic gonadotropin. Two mature fertile bucks were used for breeding the does. The goats were maintained in a free-stall barn and kept under the *Laboratory Animal Control Guidelines* of Qassim University, which basically conform to the *Guide for the Care and Use of Laboratory Animals* of the National Institutes of Health in the USA (NIH publications No. 86 to 23, revised 1996).

### 2.2. Blood Sampling Protocol

Three jugular blood samples were collected from each goat 3 wk before expected parturition (*T* −3), within 12 h of parturition (*T* 0), and 3 wk after parturition (*T* +3). Blood samples were centrifuged at 1200 ×g for 15 min, and the serum samples obtained were aliquotted in tubes.

### 2.3. Bone Metabolism Biomarker Assays

The bone biomarkers OC, b-ALP, and PYD serum concentrations were determined in the serum using commercial immunoassay kits (Quidel Corp., CA, USA) as our group described recently [17–20]. The limit of quantification of OC ranged from 2 to 32 ng/mL, and precision CVs within runs and between runs were 5–10%. The dynamic range of b-ALP was from 2 to 140 U/L, and the precision CVs within and between runs were 4–6% and 5–8%, respectively. The dynamic range of PYD was from 15 to 750 nM/L, and the precision CVs within and between runs were 6–10% and 3–11%, respectively.

### 2.4. Statistical Analysis

Data are presented as medians ± standard deviations. Statistical analysis was done with the SPSS statistics package [21]. Data normality was tested using the Kolmogorov–Smirnov test. Because data were not distributed normally, a nonparametric method (Kruskal–Wallis) was used to test the significance. Post hoc multiple comparisons among the time points *T* −3, *T* 0, and *T* +3 were then carried out using the Dunnett test. The significance value was set at *P* < 0.05.

## 3. Results and Discussion

Pregnancy and lactation are periods of significant influence on bone metabolism. Due to changes in the endocrine status and an increased need for minerals during these two periods, significant changes in mineral metabolism occur [12]. In women and rodents, late pregnancy and lactation significantly influence the extent of bone remodeling [22]. The rate of bone remodeling could be estimated indirectly, by measuring the concentrations/activities of the bone remodeling markers in the blood or urine, which are indicators of the activity of bone cells [23]. To the author's knowledge, this is the first study to evaluate the influence of the periparturient period on the serum concentrations of bone metabolism biomarkers in goats.

**Figure 1:**
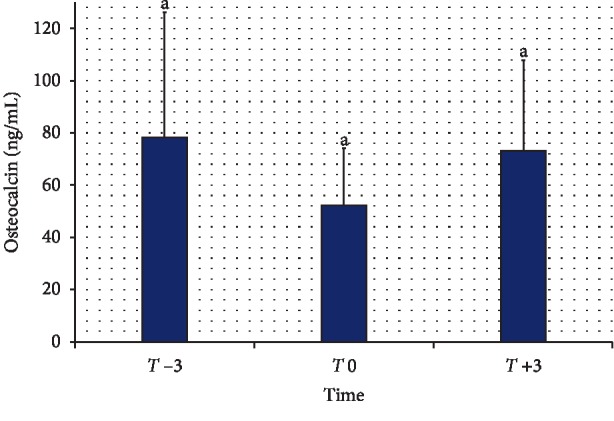
Serum concentrations of osteocalcin in goats during the periparturient period. *T* −3, 3 wk before expected parturition; *T* 0, within 12 h of parturition; *T* +3, 3 wk after parturition. Values with same letters did not differ significantly (*P* > 0.5).

**Figure 2:**
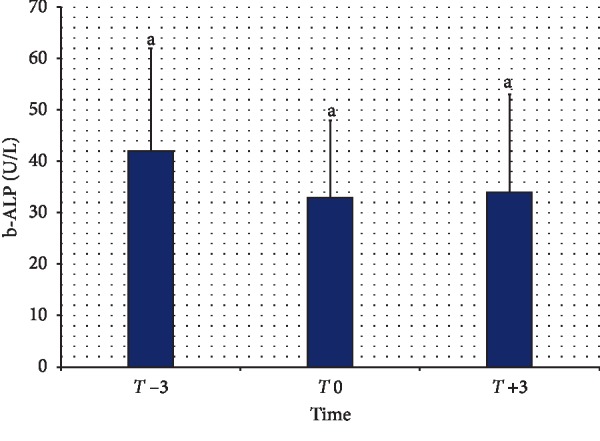
Serum concentrations of bone-specific alkaline phosphatase (b-ALP) in goats during the periparturient period. *T* −3, 3 wk before expected parturition; *T* 0, within 12 h of parturition; *T* +3, 3 wk after parturition. Values with same letters did not differ significantly (*P* > 0.5).

The serum concentrations of OC at *T* −3, T0, and *T* +3 are shown in Figure 1. Compared to a value of 77.67 ± 47.6 ng/mL at *T* −3, the serum concentrations of OC measured 51.91 ± 22.09 ng/mL at *T* 0 and 72.61 ± 35.21 ng/mL at *T* +3. A comparison of OC values at *T* −3, *T* 0, and *T* +3 did not reveal any significant difference (*P* > 0.05).  Figure 2 summarizes the serum concentrations of b-ALP at *T* −3, *T* 0, and *T* +3. Compared to a value of 42.00 ± 19.50 U/L at *T* −3, the serum concentration of b-ALP measured 32.49 ± 15.41 U/L at *T* 0 and 34.31 ± 18.89 U/L at *T* +3. A comparison of b-ALP values at *T* −3, *T* 0, and *T* +3 did not reveal any significant difference (*P* > 0.05).

The present investigations found that the bone formation biomarkers (OC and b-ALP) did not change significantly during the periparturient period of the goats. This is in agreement with data in dairy cows, where significant changes in the activity of b-ALP in the blood serum were not established during late pregnancy and early lactation [24]. On the other hand, the activity of bone formation b-ALP in the serum of mares was lower in late pregnancy than in early lactation [12].

**Figure 3:**
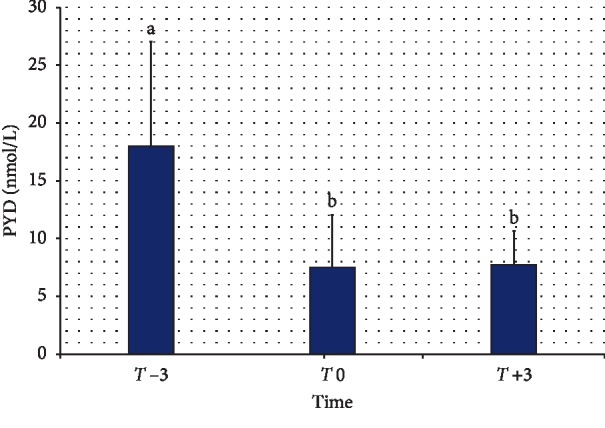
Serum concentrations of pyridinoline cross-links (PYD) in goats during the periparturient period. *T* −3, 3 wk before expected parturition; *T* 0, within 12 h of parturition; *T* +3, 3 wk after parturition. Values with different letters did not differ significantly (*P* > 0.5).

The serum concentrations of PYD at *T* −3, T0, and *T* +3 are summarized in Figure 3. Compared to a value of 17.86 ± 9.15 nmol/L at *T* −3, the serum concentration of PYD decreased significantly at *T* 0 to a value of 7.48 ± 4.50 nmol/L (*P* < 0.0001). At *T* +3, the PYD serum concentration measured 7.72 ± 2.91 nmol/L, which differed significantly from *T* −3 values (*P* < 0.05).

Concerning the bone resorption biomarker (PYD), the data in this study indicated that PYD decreased significantly at parturition compared to 3 wk before parturition. The decreases continued significantly at 3 wk after parturition. In mares, the concentrations of PYD in the blood plasma significantly increased around Day 20 after foaling, indicating an increased rate of bone resorption [12].

A significant decrease of estrogen in the goats at 3 wk after parturition has been reported in our previous study [16]. These results are therefore consistent with a report in lactating women and another in mares, where low levels of estradiol were reported to influence the rate of bone turnover [12, 22, 24]. Filipovic et al. [12] suggested that high concentrations of estradiol in the blood during late pregnancy could cause a decrease in the number of osteoblasts because estrogens suppress the self-renewal of osteoblast progenitors, while their antiosteoclastogenic effects are thought to be secondary to impaired osteoblast formation [25]. Hence, the lower concentrations of estradiol in the blood after parturition could contribute to an elevated bone resorption rate.

The present findings suggest that decreasing estrogen levels postpartum may enhance osteoclast activity that, in turn, would increase bone resorption. It has been reported that a cyclical variation in bone turnover occurs over the course of the estrous cycle in postpartum dairy cows, with decreases in plasma estrogen below a critical threshold correlating with enhanced bone resorption [26]. The same author also reported that alterations in the concentration of the bone resorption marker TRAP5b negatively correlated with estrogen levels; enhanced TRAP5b activity correlated with decreased estrogen concentrations below a defined level. This finding is consistent with the results of our previous study [16], where the estrogen level was increased at parturition while bone resorption was decreased. In mares, there was a fluctuation in the bone turnover rate during the estrous cycle; serum concentrations of the markers of bone formation and bone resorption were increased during the luteal phase compared with mares at other stages of the estrous cycle [27]. The combined results of increased bone resorption at parturition in this study together with the increased estrogen concentration somewhat mirror the finding of increased serum activity of the bone resorption marker TRAP5b in postmenopausal women and the subsequent decreases in its activity in women given estrogen replacement therapy [28].

## 4. Conclusions

The results of this study showed that the bone formation biomarkers did not change significantly during the periparturient period, while the bone resorption biomarker decreased significantly at parturition compared to 3 wk pre- and postparturition. The significantly increased serum estrogen around parturition may have had a role in the increased bone resorption at this time. A limitation of this study was the use of only PYD biomarker to declare the bone resorption status. It would be more beneficial if TRAP5b or other resorption biomarkers could be estimated.

## Data Availability

The data used to support the findings of this study are included within the article.
